# Functional magnetic resonance imaging and brain functional connectivity in migraine

**DOI:** 10.1186/1129-2377-16-S1-A11

**Published:** 2015-09-28

**Authors:** Antonio Russo, Gioacchino Tedeschi, Alessandro Tessitore

**Affiliations:** Department of Medical, Surgical, Neurological, Metabolic and Aging Sciences, Second University of Naples, Naples, Italy; MRI Research Center SUN-FISM, Second University of Naples, Naples, Italy; Institute for Diagnosis and Care “Hermitage Capodimonte”’, Naples, Italy

The human brain is a network of a large number of different brain regions characterized by specific task and function, but which are continuously sharing information with each other. Functional communication between brain regions is likely to play a key role in complex brain processes, thriving on the continuous integration of information across different regions of the brain. This makes the examination of brain functional connectivity (FC) of high importance, providing new insights in the human brain organization.

In the context of functional neuroimaging, FC is related to the relationship between the neuronal activation patterns of anatomically separated brain regions, reflecting the level of functional communication between regions. During resting-state (RS) studies, volunteers were instructed to relax while their level of spontaneous brain activity was measured throughout the period of the experiment. Biswal and colleagues were the first to demonstrate that during rest the left and right hemispheric regions of the primary motor network are not silent, but show a high correlation between their fMRI BOLD time-series, suggesting ongoing information processing and ongoing FC between these regions during rest. Several studies have replicated these pioneering results, showing a high level of FC also between regions of other known functional networks. In fact, the spontaneous BOLD signal fluctuations are topographically organized in highly reproducible functional networks, called RS networks (RSNs). The most commonly reported RSNs are the default mode network (DMN), the fronto-parietal (or executive) network (FPN), the sensorimotor network (SMN) and the visual and auditory networks. Several RS-fMRI studies have recently demonstrated disrupted RSNs FC in patients suffering from chronic pain conditions suggesting that pain has a widespread impact on overall brain FC. More recently, RS-fMRI has been applied in studies focused on migraine, to assess alterations of baseline intrinsic brain activity, likely related to long term migraine attacks. Along this research line, several studies have analysed the alteration of baseline FC within different RSNs in both patients with migraine without aura and migraine with aura. All this body of scientific data has to be interpreted with caution because of small sample sizes, patients’ clinical heterogeneity and sometimes lack of consistent methodological approach. However, the interpretation of the biological significance of these various FC changes could remain incomplete without a combination of expanding genomic information about neurochemical pathways and genetic polymorphisms linked to specific migraine phenotypes or subtypes.

